# Label-Free Rapid Quantification of Abscisic Acid in Xylem Sap Samples Using Surface Plasmon Resonance

**DOI:** 10.3390/bios15110725

**Published:** 2025-11-01

**Authors:** Laurien Volkaert, Sam Noppen, Veronika Turečková, Ondřej Novák, Dominique Schols, Jeroen Lammertyn, Bram Van de Poel, Dragana Spasic

**Affiliations:** 1 Molecular Plant Hormone Physiology Lab, KU Leuven, Willem de Croylaan 42, 3001 Leuven, Belgium; laurien.volkaert@kuleuven.be; 2 Biosensors Group—Lammertyn Lab, KU Leuven, Willem de Croylaan 42, 3001 Leuven, Belgium; dragana.spasic@kuleuven.be; 3 KU Leuven Plant Institute (LPI), Kasteelpark Arenberg 31, 3001 Leuven, Belgium; 4 Molecular, Structural and Translational Virology, Rega Institute, KU Leuven, Herestraat 49, 3000 Leuven, Belgium; sam.noppen@kuleuven.be (S.N.); dominique.schols@kuleuven.be (D.S.); 5 Laboratory of Growth Regulators, Institute of Experimental Botany of the Czech Academy of Sciences, Palacký University, Šlechtitelů 27, 783 71 Olomouc, Czech Republic; veronika.tureckova@upol.cz (V.T.); ondrej.novak@upol.cz (O.N.)

**Keywords:** abiotic stress, abscisic acid, direct SPR assay, drought stress, high salinity stress, label-free, non-competitive immunoassay, tomato plant, UPLC-MS/MS, xylem sap samples

## Abstract

The phytohormone abscisic acid (ABA) plays a central role in organizing adaptive responses in plants to various abiotic stresses, helping the plant minimize the negative impact on growth and development. Rapid and direct detection of ABA is valuable for investigating plant responses to abiotic stress. In this work, we propose a novel label-free, non-competitive immunoassay for detecting and quantifying ABA easily and rapidly using a surface plasmon resonance (SPR) biosensor. The SPR sensor chip was functionalized with a commercial anti-ABA antibody, characterized for its affinity, binding kinetics, and specificity using the same platform. The direct assay demonstrated high specificity and sensitivity, with a calculated limit of detection of 1.36 ng/mL in buffer. The new immunosensor was applied to determine ABA concentrations directly in xylem sap samples from tomato plants subjected to abiotic stress (drought and high salinity) and was able to accurately reflect ABA levels corresponding to the applied stress. The results were comparable to the reference method, ultra-performance liquid chromatography coupled with tandem mass spectrometry (UPLC-MS/MS), establishing this new immunosensor as a novel detection method for rapid and reliable monitoring of ABA levels associated with abiotic stress in tomato plants.

## 1. Introduction

Plants are experiencing environmental stresses more than ever due to the ongoing climate change. Both plant abiotic and biotic stresses affect plants, of which abiotic stresses caused by heatwaves, drought, heavy precipitation, and tropical cyclones are occurring more frequently due to the rise in temperature of the atmosphere, land, and ocean [1]. In addition, biotic stresses are also being exacerbated by climate change, since the development, spread, population dynamics, and the interactions with natural enemies of agricultural insect pests and diseases are affected [2]. These biotic and abiotic stresses negatively impact plant growth, development, and metabolism, consequently impacting crop yield. To maintain proper growth under these conditions, plants use several response strategies, including physiological, biochemical, and molecular mechanisms [3,4], with phytohormones playing an important role. Salicylic acid and jasmonic acid are both involved in defense responses against biotic stresses such as pathogens and herbivorous insects [5], while abscisic acid (ABA), ethylene, auxin, and cytokinin primarily regulate plant responses in the face of harmful environmental conditions [6,7].

The phytohormone ABA plays a central role in organizing adaptive responses in plants to various abiotic stresses, such as drought and salinity. Under drought stress, ABA accumulates in the guard cells, inducing stomatal closure to minimize water loss through transpiration [8]. To protect against salt stress, ABA promotes the production of osmolytes, such as e.g., proline, to maintain cell turgor and stabilize membranes [9]. ABA has a broad crosstalk with other phytohormones to regulate crucial functions of plants during adverse conditions [10,11]. In addition to protecting against external stresses, ABA regulates various physiological processes, including seed dormancy and germination. To prevent germination during unfavorable conditions, ABA is crucial in inducing and maintaining seed dormancy [12,13]. Considering all these important roles of ABA, rapid and direct detection of ABA levels can be used as an early stress-diagnostic tool or a method to study ABA’s involvement in plant development.

Current methods to measure ABA concentrations mostly rely on liquid chromatography (LC) and, less frequently, on gas chromatography (GC) coupled with tandem mass spectrometry detection (MS/MS) [14,15,16,17,18,19,20,21]. Although these classic analytical techniques are considered to be the industry standard for measuring ABA due to their high sensitivity, selectivity, and versatility for identifying and quantifying various small molecules, they require complex sample preparation and skilled technicians to operate the advanced instrumentation and consume a high volume of organic solvent. These analyses are also not very accessible due to the expensive equipment with high purchase, maintenance, and operational costs. Furthermore, only a few laboratories are equipped to perform these hormone analyses for research, giving rise to high costs per sample and long waiting times for obtaining results.

More traditional methods for determining ABA concentrations are immunoassays, such as radioimmunoassays and enzyme-linked immunosorbent assays (ELISA) [22,23]. For instance, to examine the effects of short photoperiods and low-temperature stress on ABA production, Pagter et al. determined fluctuations in ABA concentrations in unpurified xylem sap with an ELISA using a monoclonal anti-ABA antibody [24]. While laboratory-developed ELISAs are common in scientific research, commercial ABA ELISA kits are also broadly available and have been used by several research groups to determine the ABA concentrations in plants [25,26,27]. Previously reported ELISA examples are all based on a competitive, indirect detection method and show that this technique is sensitive enough to detect ABA. However, although ELISA is more accessible than the previously mentioned LC-MS/MS technique, it offers lower detection sensitivity and requires significantly higher sample volumes.

In recent years, significant advances have been made in the development of biosensors for the detection of ABA. These approaches primarily differ in their signal transduction mechanisms, including electrochemical biosensors [28,29] and optical biosensors, which are based on fluorescence [30,31] or luminescence [32]. While these techniques have demonstrated promising sensitivity and potential for rapid or portable analysis, they often face challenges related to matrix interference or the requirement for genetically modified plants.

To address the disadvantages of the previously mentioned techniques, we developed for the first time an immunoassay for rapid (two minutes) and direct (label-free) ABA quantification that is more sensitive than traditional ELISAs but of significantly lower complexity compared to the LC- and GC-MS/MS. In this context, we used the Biacore™ system [33], a commercially available surface plasmon resonance (SPR) platform considered to be the gold standard in the field that has been frequently used in medical diagnostics and pharmaceutical development, environmental monitoring and protection, as well as food quality control [34,35,36]. Although Biacore™ has been used to study ABA binding/interactions to e.g., its receptor pyrabactin resistant 1 like 1 (PYL1), and the downstream protein phosphatase 2C (PP2C) abscisic acid insensitive 1 (ABI1) [37,38,39,40], it has not been used as a diagnostic tool to determine ABA concentration levels. To achieve the latter, we first characterize a commercial monoclonal anti-ABA antibody by measuring its affinity and binding kinetics against ABA and an ABA-BSA conjugate, used to develop the antibody, followed by cross-reactivity testing against ABA analogues. Next, we develop and optimize a direct label-free SPR immunoassay to quantify ABA concentration levels in xylem sap samples of tomato plants facing abiotic stress. Importantly, we also benchmark our Biacore™ method against the gold standard reference technology, UPLC-MS/MS. This new SPR immunosensor has the potential to become a useful monitoring tool capable of tracking dynamic changes in ABA levels in plants, opening up opportunities for more in-depth studies of the regulatory mechanisms of ABA. Furthermore, the bioassay optimized on the SPR Biacore™ system could be adapted to other, more accessible SPR-based biosensing platforms, thereby providing alternatives to costly analytical techniques.

## 2. Materials and Methods

### 2.1. Reagents and Materials

Abscisic acid, 98% (ABA, cat. no: 342401000), ABA bovine serum albumin (ABA-BSA, cat. no: CSB-MC00421b0105), ABA precursor abscisic aldehyde (ABA aldehyde, cat. no: SC-479414), β-D-glucopyranosyl abscisate (ABA glucosyl ester, ABA-GE, cat. no: HY-111974-1) and 1-aminocyclopropane-1-carboxylic acid (ACC, cat. no: A1178) were purchased from Thermo Fisher Scientific (Waltham, MA, USA), Cusabio (Houston, TX, USA), Bio-Connect (Huissen, The Netherlands), Sanbio B.V. (Uden, The Netherlands) and TCI (Tokyo, Japan), respectively. Bovine serum albumin (BSA, 9998) was purchased from Cell Signaling Technology (Danvers, MA, USA). The mouse anti-ABA monoclonal antibody (cat. no: MBS7041609) was acquired from MyBioSource (San Diego, CA, USA). Sodium acetate and Tween^®^20 were purchased from Merck/Sigma-Aldrich (Darmstadt, Germany). NaCl and 2-(4-(2-hydroxyethyl)-1-piperazinyl)ethanesulfonic acid (HEPES) were purchased from Carl Roth (Karlsruhe, Germany), whereas dimethyl sulfoxide (DMSO) was acquired from Thermo Fisher Scientific. N-hydroxysuccinimide (NHS), 1-ethyl-3-(3-dimethylaminopropyl)carbodiimide (EDC), 10 mM of sodium acetate, pH 5.0, 1 M of ethanolamine, pH 8.0, and HCl, pH 1.5, were obtained from Cytiva (Uppsala, Sweden) as well as CM5 sensor chips and the Biacore™ 8K instrument. Polypropylene 96-well plates (651201) used to deposit samples for Biacore™ experiments were acquired from Greiner Bio-One (Kremsmünster, Austria).

### 2.2. Plant Material, Growth Conditions, and Xylem Sap Sampling

Tomato (*Solanum lycopersicum*, var. Ailsa Craig) seeds were sown in soil in growing trays and moistened with deionized water. Approximately two weeks after germination, the seedlings were transplanted onto individual rockwool blocks and watered with nutrient solution (electrical conductivity (EC): 2.5–3 dS/m, pH: 5.5–6; [41]). The plants were grown in a climate-controlled growth chamber with a set temperature of 26 °C and a constant relative humidity of 55%. The plants were grown under a long-day photoperiod with 16 h of light and 8 h in the dark, and a light intensity at the shelf level of 150 µmol/(m^2^s^1^) (VS12 LED, Sunritek, Shenzhen, China).

The plants were sampled once they reached leaf stage eight (BBCH 18 [42]). The xylem sap sampling method consisted of cutting the top part of the plant close to the surface, right below the cotyledons. The sampling of xylem sap relied on active root pressure pushing out the sap and forming droplets on top of the cut stem. The first droplet was discarded due to a possible contamination of broken cells caused by cutting the stem. The sap was collected by pipetting it off the stem and depositing it in Eppendorf tubes placed on ice. This process was repeated for one hour, stopping before the wound response could set in and alter the xylem sap matrix. After collection, the tubes were stored at −20 °C.

### 2.3. Abiotic Stress Treatments

Twenty plants at leaf stage eight were used for the stress experiments. The drought and salt stress experiments each included ten plants, with five plants receiving the stress treatment and five plants serving as controls. Until the start of the abiotic stress, both treatment groups received the same nutrient solution with an EC of 2.5–3 dS/m and a pH of 5.5–6. For the drought treatment, the treated plants were not irrigated anymore, while the control plants continued to be irrigated throughout the experiment. For the salt stress treatment, the control nutrient solution (EC 2.5–3 dS/m) was supplemented with NaCl to reach severe salinity (EC 10 dS/m) according to Holsteens et al. [41], whereas the control plants continued to receive the original nutrient solution. For both stress treatments, the abiotic stress period lasted for 48 h, after which the sampling method described above was employed to collect the xylem sap. Each sample was considered a biological replicate, without pooling the samples.

### 2.4. Development of a Direct Label-Free SPR Immunoassay

#### 2.4.1. Biacore™ CM5 Sensor Chip Functionalization with Anti-ABA Antibody

All experiments were performed on a CM5 sensor chip docked inside a Biacore™ 8K system instrument. The Biacore™ 8k flow system has eight separate channels on the sensor surface, each containing two flow cells (Fc): Fc1 being the reference cell (functionalized using the same coupling strategy but without the ligand) and Fc2 being the active cell with ligand. The signal generated in Fc1 is used to correct for any possible non-specific binding of the matrix to the sensor chip and for bulk refractive index (RI) differences between the sample and the flow buffer. To functionalize the sensor surface, the ligand, in this case, the anti-ABA antibody, was covalently coupled using amine coupling chemistry [43]. Briefly, a sensor chip was docked in the Biacore™ 8K system and equilibrated in 10 mM HEPES buffer, supplemented with 150 mM NaCl and 0.05% (*v*/*v*) Tween^®^ 20 (pH 7.4), and washed with 1 M NaCl in 50 mM NaOH. Next, the sensor surface was activated for seven minutes in a 1:1 (*v*/*v*) mixture containing NHS (0.05 M) and EDC (0.2 M) at a flow rate of 10 µL/min. Subsequently, the anti-ABA antibody (20 µg/mL in 10 mM of sodium acetate, pH 5.5) was immobilized for seven minutes until saturation was reached. Finally, deactivation of carboxyl groups was done using ethanolamine for seven minutes at 10 µL/min.

#### 2.4.2. Anti-ABA Antibody Characterization Using the Biacore™ SPR Platform

All characterization experiments on the Biacore™ platform were performed at 25 °C in running buffer (100 mM of acetate buffer + 150 mM of NaCl + 0.05% (*v*/*v*) Tween^®^ 20, pH 7). Serial dilutions of ABA (1.37–1000 ng/mL in a three-fold dilution), ABA-BSA or BSA (31.3–4000 ng/mL in a two-fold dilution) were injected for 120 s at a high flow rate of 30 μL/min to ensure enough analyte is transported to the surface by diffusion (different concentration ranges were selected for ABA and ABA-BSA due to the significant difference in their molecular weights, being 264 Da and 66.5 kDa, respectively). The dissociation was monitored for five minutes. No regeneration of the surface was needed for ABA, whereas for ABA-BSA and BSA, a regeneration step using HCl pH 1.5 was included due to an incomplete dissociation of ABA-BSA from the sensor chip. Double referencing was implemented through the use of the reference flow cell (Fc1), as well as buffer blanks, the latter accounted for possible baseline drift and injection noise. Both signals were then subtracted from the original signal to focus on the actual binding signal of the ABA (or ABA-BSA) to the antibody and to produce high-quality data for further analysis. The equilibrium dissociation constant (affinity) (K_D_) and kinetic rate constants (k_a_, k_d_) were derived after fitting the experimental data to the 1:1 Langmuir binding model using the Biacore™ Insight Evaluation Software v6.0. To measure the affinity and kinetics of anti-ABA antibody binding to ABA analogues, the running buffer was supplemented with 2% DMSO, which was required for their dissolution. A DMSO concentration series was included to remove the DMSO effect on the measured responses [44].

#### 2.4.3. Development of an ABA Calibration Curve in Running Buffer

An ABA calibration curve was generated using a running buffer spiked with a two-fold dilution series of ABA (2–1024 ng/mL). To prevent nonspecific binding from the xylem sap matrix, BSA (1 mg/mL) was added to the running buffer and xylem samples as a blocking agent for the chip surface. The blocking was executed before sample injection. All samples were injected at a low flow rate of 10 µL/min for 120 s to minimize sample consumption. Dissociation was monitored for six minutes. An additional wash step with 20% isopropanol in 40 mM NaOH was used to keep the flow system clean. At the report point in the dissociation phase, 10 s after the end of sample injection, the binding response was measured in running buffer to avoid bulk RI contributions from the xylem sap matrix during sample injection. Calibration curves were generated by plotting the binding responses in response units (RUs) of the calibration samples against the corresponding concentrations of ABA, and fitting a four-parameter logistic regression in the Biacore™ Insight Evaluation Software v6.0. The LOD was defined as the mean blank value plus three times the standard deviation (SD) of replicate measurements on blank samples [45].

### 2.5. Quantification of ABA in Xylem Sap Samples from Abiotic Stress Plants

The quantification of ABA in xylem sap samples using the Biacore™ SPR immunoassay was performed based on the established ABA calibration curve in buffer, by following the corresponding Biacore™ application guide [45]. The xylem sap samples, collected from plants subjected to abiotic stress, were centrifuged at 2000× *g* for one minute and diluted two-fold in running buffer. These samples were analyzed following the same conditions and protocol as explained in Section 2.4. Reference and blank subtraction were not applied for these measurements. Although a reference surface (Fc1) is highly valuable in many SPR applications, it is not recommended for concentration measurements, as errors can be caused due to the difference in nonspecific binding of the sample matrix between the active (Fc2) and reference (Fc1) surface [45]. Additionally, blank xylem subtraction was not possible either because xylem sap samples collected from control plants also contain endogenous ABA.

The UPLC-MS/MS method, as described by Turečková et al. [21] with some modifications, was used as a benchmark to quantify endogenous levels of ABA and its catabolites in the matching tomato plant xylem sap samples from the Biacore™ SPR immunoassay experiments. Briefly, tomato xylem sap was homogenized using a vortex, and 5 μL of each sample was transferred into vials, then spiked with 3 μL (3 pmol) of a labelled internal standard mixture (including (−)-7’,7’,7’-^2^H_3_-phaseic acid; (−)-7’,7’,7’-^2^H_3_-dihydrophaseic acid; (-)-8’,8’,8’-^2^H_3_-neophaseic acid; (+)-4,5,8’,8’,8’-^2^H_5_-ABAGE; (−)-5,8’,8’,8’-^2^H_4_-7’-OH-ABA and (+)-3’,5’,5’,7’,7’,7’-^2^H_6_-ABA). The samples were then diluted with 30% (*v*/*v*) methanol up to a final volume of 30 μL. A 3 μL aliquot of each sample was injected onto a reversed-phase column (Acquity UPLC BEH C18, 1.7 μm, 2.1 × 150 mm, Waters, Milford, MA, USA) and analyzed by UPLC-MS/MS using a Xevo TQ-XS triple quadrupole mass spectrometer (Waters).

### 2.6. Statistical Analysis

All data were analyzed and visualized using GraphPad (Version 8.0.2, Delta Prism). For all experiments, due to small sample sizes, the normality of the residuals was tested using both the D’Agostino-Pearson omnibus K^2^ and the Shapiro–Wilk W normality tests [46,47]. In the first analysis, the RU signal values of both ABA analogues were individually compared to those of ABA in running buffer supplemented with 2% DMSO (n = 4). None of the data sets passed the normality tests, so the significant differences were confirmed by a Mann–Whitney *U* test for the distribution-free assumption (*p*-value < 0.05) [48]. In the second analysis, the ABA concentration measurements of the abiotic stress-treated plant samples were compared with their respective controls. All data sets were confirmed to pass both normality tests except for the high salinity data set with the Biacore™ platform and with the UPLC-MS/MS. For reasons of uniformity, the significant difference for all data sets was confirmed by a Mann–Whitney *U* test for the distribution-free assumption (*p*-value < 0.05). In the third analysis, correlation plots were constructed between the Biacore™ and the UPLC-MS/MS techniques. All data sets passed the normality tests, so the Pearson correlation coefficient was used to compute the correlation between the two techniques (*p*-value < 0.05) [49].

## 3. Results

### 3.1. Anti-ABA Antibody Characterization Using the Biacore™ SPR Platform

**Figure 1:**
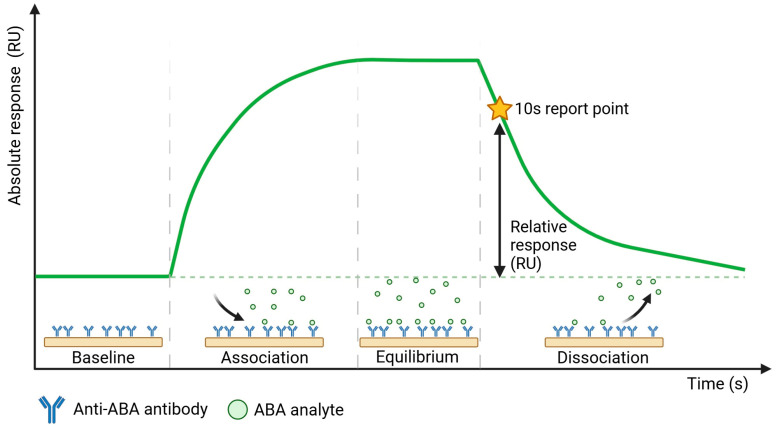
Schematic representation of the sensorgram obtained from the direct SPR immunoassay performed on the Biacore™ platform.

To establish sensitive and specific detection of ABA in xylem sap, we first characterized the selected commercial anti-ABA antibody (ligand) for its interaction with the target (analyte) molecule. This was performed using the Biacore™ SPR platform, which enables the study of these interactions in a direct manner even when target molecules are as small as ABA (264.32 g/mol). In addition to ABA alone, we also included ABA-BSA in this characterization study, as this conjugate was used for the development of the selected antibody (since ABA alone is too small to elicit an immune response), and BSA as a negative control. Anti-ABA antibody was covalently immobilized on the sensor chip via amine coupling, and multiple concentrations of either ABA, ABA-BSA, or BSA were flown over this sensor chip. A schematic representation of the sensorgram obtained from such direct SPR immunoassay with ABA is shown in Figure 1, revealing four stages of a sample measurement: (1) the baseline, established in the running buffer; (2) the association phase, starting upon sample injection that shows ABA binding to the anti-ABA antibody in real time; (3) binding equilibrium and (4) dissociation of the bound ABA that takes place in the running buffer. The ABA response binding levels (RUs) were measured at a report point during the dissociation phase when only running buffer flowed over the sensor surface, 10 s after the end of sample injection, to avoid bulk RI contributions from the sample matrix, such as the xylem sap. In this context, it is important to note that data shown in Supplementary Figure S1 indicates the absence of nonspecific binding of the xylem sap matrix to the reference channel Fc1, either left blank or functionalized with an unrelated antibody to mimic the surface characteristics of the anti-ABA antibody functionalized surface of Fc2. Therefore, the obtained SPR signal originates only from bound ABA.

**Figure 2:**
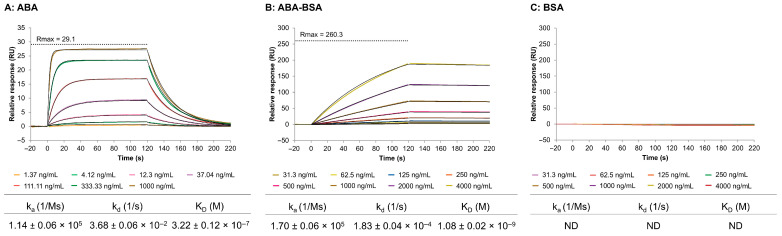
SPR sensorgrams (colored lines) and fitted curves (black lines) showing the interaction of the anti-ABA antibody with ABA (**A**), ABA-BSA (**B**), and BSA (**C**). Seven ABA concentrations (1.37–1000 ng/mL in a three-fold dilution) and eight ABA-BSA and BSA concentrations (31.3–4000 ng/mL in a two-fold dilution) were tested. The tables below the graphs indicate the kinetic rate constants (k_a_ and k_d_) and the average K_D_ value ± SD of the 1:1 binding model (n = 3). ND = not detectable.

The actual SPR sensorgrams obtained from the direct interaction of anti-ABA antibody, immobilized on the chip surface, with ABA, ABA-BSA, and BSA are depicted in Figure 2A, B, C, respectively. The results are shown from one representative channel out of eight, after correction using the signal from Fc1 and the buffer blank. The kinetic rate constants (k_a_ and k_d_) and the equilibrium dissociation constant (K_D_) were derived by fitting the experimental data to the 1:1 Langmuir binding model [50]. The anti-ABA immobilization graph and the residuals graph for the ABA, ABA-BSA, and BSA sensorgrams are all provided in Supplementary Figure S2. Higher ABA concentrations resulted in a noticeable increase in signal due to the binding of ABA to the anti-ABA antibody on the surface. As can be seen from the sensorgram shape, the binding is transient and characterized by a fast association and dissociation phase. Importantly, a dissociation phase of two minutes was enough to remove all ABA from the anti-ABA antibody surface, which avoided the use of surface regeneration. The corresponding k_a_ and k_d_ values, as well as K_D_, are reported in the table under Figure 2A. The dissociation constant K_D_ of 3.22 ± 0.12 × 10^−7^ M indicated a moderate affinity.

The interaction of ABA-BSA with the anti-ABA antibody produced a visually different sensorgram with slower binding kinetics (Figure 2B). The response signal was notably higher for ABA-BSA, which can be explained by its significantly higher molecular mass (66.5 kDa) compared to ABA (264 Da). When translated into molar concentrations, the maximum ABA concentration (i.e., 1000 ng/mL) corresponds to 3780 nM, whereas the maximum ABA-BSA concentration (i.e., 4000 ng/mL) corresponds only to 60.15 nM. Therefore, even though lower molecular concentrations were used for ABA-BSA, its larger mass resulted in higher response signals than ABA. Interestingly, there was no equilibrium plateau forming, nor any dissociation after injection, indicating the need for a regeneration step. The K_D_ value for ABA-BSA was also a hundred times lower than the K_D_ for ABA, due to the k_d_ value being a hundred times smaller, suggesting a strong interaction of the antibody with the ABA target in the nanomolar range. This observation can be explained by the fact that the anti-ABA antibody was raised against ABA-BSA. Importantly, the negative control experiment using BSA as analyte confirmed that the antibody response to ABA-BSA is due to the specific recognition of ABA and not due to nonspecific binding to the carrier protein BSA.

The specificity of the anti-ABA antibody towards ABA was further investigated through additional cross-reactivity studies with ABA analogues (i.e., namely ABA-related compounds, including the precursor ABA aldehyde and the glucose conjugate ABA-GE) and another small plant molecule, ACC (a precursor of the plant hormone ethylene). Experiments involving ABA and its analogues were conducted in running buffer supplemented with 2% DMSO, which was required to dissolve the analogues (the DMSO bulk shift corrections are presented in Supplementary Figure S3). In contrast, ACC was analyzed using the original running buffer. From the direct immunoassay performed as described above, the binding kinetic parameters were determined for ABA, ABA aldehyde, ABA-GE, and ACC as listed in Table 1. The results show that the anti-ABA antibody binds most strongly to its native target ABA (K_D_: 4.66 ± 0.73 × 10^−7^ M) while exhibiting considerably lower affinity towards ABA aldehyde (K_D_: 8.12 ± 0.83 × 10^−6^ M) and ABA-GE (K_D_: 1.74 ± 0.31 × 10^−5^ M). Additionally, when all four were injected at a consistent concentration of around 150 ng/mL, both ABA aldehyde and ABA-GE produced significantly lower RU values on the SPR platform, in contrast to ABA. The negative control, ACC, showed no detectable interaction with the antibody under the tested conditions and, thus, none of the parameters could be determined. Kinetic binding curves with Rmax value and the related residual for each of the analytes are presented in Supplementary Figure S4.

### 3.2. Development of a Direct Label-Free SPR Immunoassay

**Figure 3:**
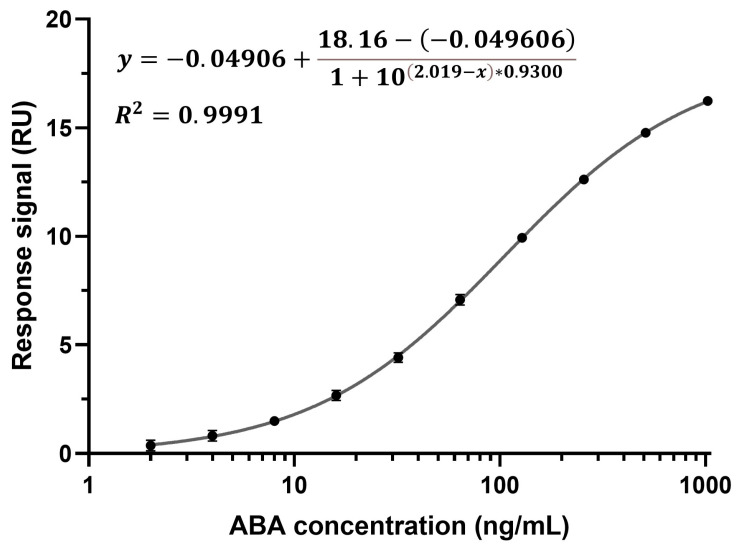
Calibration curve from channel one of ABA spiked in running buffer with added BSA, detected at a range of concentrations (2–1024 ng/mL using a two-fold dilution) with the direct, non-competitive immunoassay (n = 3, average value with one SD, too small to be distinguishable on the graph, LOD = 1.36 ng/mL).

Calibration curves were established in all eight channels of the CM5 chip by detecting ABA spiked into running buffer at concentrations ranging from 2 to 1024 ng/mL using a two-fold dilution (Figure 3 depicts a calibration curve from channel one). All ten concentrations were measured from low to high and in triplicate (n = 3). Due to the rapid k_d_, no regeneration condition was required between cycles. The running buffer was supplemented with BSA to block the chip surface. The binding characteristics of ABA diluted in running buffer and ABA diluted in running buffer supplemented with BSA were found to be identical, indicating that BSA does not influence the binding of ABA to the anti-ABA antibody (Supplementary Figure S5). To build the calibration curves, the response at the report point during the dissociation phase was plotted against the corresponding ABA concentration. ABA quantification at this point, when running buffer flows over the chip surface, enables the selective detection of ABA that is specifically bound to the sensor surface. This report point eliminates matrix interference and the need for a calibration curve in the xylem sap matrix. Moreover, no Fc1 and buffer correction were performed as these calibration curves were to be used to derive the unknown ABA concentrations from xylem sap samples (see Section 3.3). A sigmoidal, four-parameter logistic regression was used to fit the calibration curve, for which the limit of detection (LOD) was calculated to be 1.36 ng/mL, which is below the lowest measured ABA concentration (2 ng/mL) [45]. It is important to note that the stability of the immunosensor chip was evaluated and found stable for at least three days and 77 cycles, with no noticeable initial decline (Supplementary Figure S6).

### 3.3. Quantification of ABA in Xylem Sap Samples from Abiotically Stressed Plants

**Figure 4:**
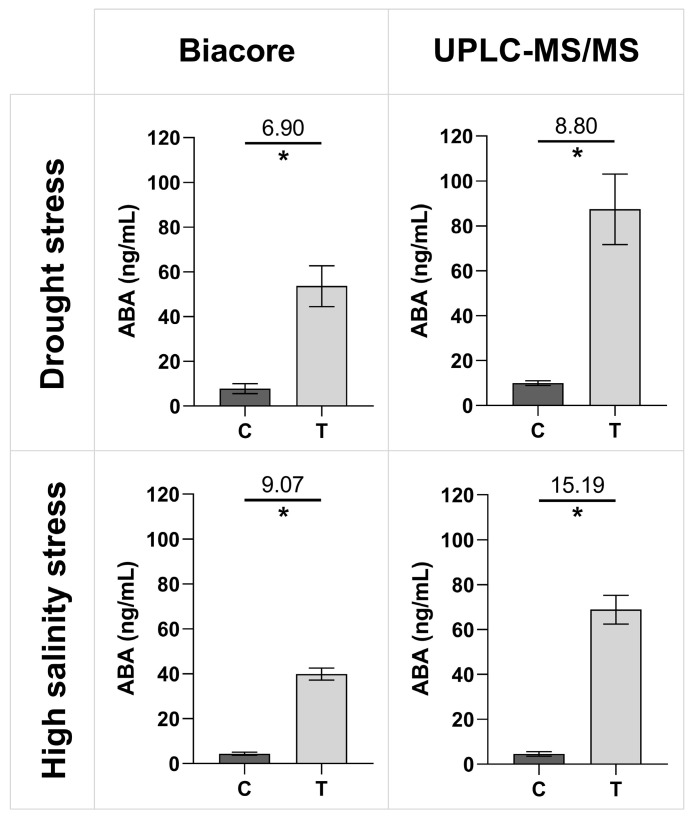
Average ABA concentrations with one SD in xylem sap samples of tomato plants treated with drought or high salinity, as quantified by the new direct SPR Biacore™ immunoassay (ng/mL) and UPLC-MS/MS (ng/mL). The fold difference between control (C) and treated plants (T) is displayed on top of the line. Significant differences were tested with a Mann–Whitney U test for each panel between the control and the treatment, indicated by the asterisk (n = 5 biological replicates, *p* < 0.05).

The established SPR Biacore™ immunoassay was further employed to quantify unknown ABA concentrations in xylem sap samples collected from control and abiotically stressed tomato plants, which had undergone drought and high salinity treatments. It is important to note that, for each tested category (i.e., control plants for drought treatment, control plants for high salinity treatment, as well as drought and high salinity treatments), the xylem sap was collected from five different plants, with each sample being considered a biological replicate. Each sample was then divided into two aliquots for parallel analysis using our newly established SPR Biacore™ immunoassay and UPLC-MS/MS. Figure 4 provides an overview of the results from the two assays, with ABA concentration values reported for both the Biacore™ SPR immunoassay and the UPLC-MS/MS. The treated plants under drought and high salinity stress exhibit a significantly higher ABA concentration than the control plants for both techniques. Additionally, the relative signal change between the stressed and control plants can be compared, defined as the ratio of the treatment signal over the control signal (displayed on top of the line). For both Biacore™ and UPLC-MS/MS, the fold changes for the high salinity treatments are higher than the fold changes for the drought treatments. When compared to each other, fold changes are slightly lower for the Biacore™ immunosensor than for UPLC-MS/MS.

**Figure 5:**
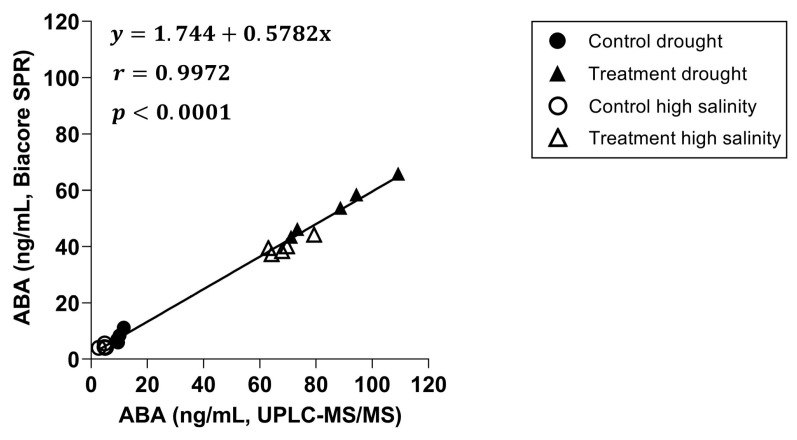
Correlation graph between Biacore™ SPR and UPLC-MS/MS with a linear regression and a Pearson correlation coefficient included (n = 20, *p* = 0.05).

Next, a correlation plot was created between both techniques (Figure 5), including a linear regression and a Pearson correlation coefficient. The slope of the linear regression with a value of 0.5782 indicates an underestimation of the ABA concentrations by the Biacore™ immunoassay compared to the UPLC-MS/MS, while the Pearson correlation coefficient with a value of 0.9972 shows a strong positive correlation between both.

## 4. Discussion

Establishing a direct, non-competitive, label-free immunoassay for the direct detection of small, low-molecular-weight molecules, such as phytohormones, remains a significant challenge. This is particularly true for SPR, as the SPR signal depends on mass changes at the sensor surface resulting from molecular binding [51,52,53]. Although Biacore™ platforms have previously been used to study ABA binding and interactions through the interaction of the PYR/PYL receptor protein family with the downstream PP2C [37,38,40], SPR technology has not yet been applied in combination with antibodies for the detection and quantification of ABA. Antibodies are more commonly employed in electrochemical biosensors for ABA detection [28,54]. In this work, we successfully developed a direct immunoassay for the small molecule ABA on the Biacore™ platform. This approach enabled detailed characterization of the antibody–antigen interaction, including determination of affinity and kinetic rate constants. The selected antibody demonstrated moderate affinity in the high nanomolar range (K_D_ = 3.22 ± 0.12 × 10^−7^ M) (Figure 2), as well as a high selectivity, indicated by a stronger binding affinity to ABA (K_D_ = 4.66 ± 0.73 × 10^−7^ M) compared to its analogues (K_D_ of 1.74 ± 0.31 × 10^−5^ M and 8.12 ± 0.83 × 10^−6^ M) (Table 1). Furthermore, the antibody was successfully immobilized onto the sensor surface, enabling direct detection of the target analyte. Compared to the more traditional competitive ELISA, this direct interaction, rather than the measurement of the reduced antibody binding through a competition reaction, is a more simplified, time- and reagent-efficient method.

This study highlights the analytical challenges associated with quantifying ABA in xylem sap, primarily due to the presence of endogenous hormone levels and matrix effects. The primary function of xylem is to transport water and minerals from root to shoot, but it also carries small quantities of phytohormones, secondary metabolites, carbohydrates, amino acids, and proteins [55,56,57,58,59,60]. Among these, ABA accumulates in root tissues, is released to the xylem vessels, and is transported to the shoot to regulate physiological processes [61,62]. This endogenous ABA, inherently present in xylem sap, will be detected by the immunosensor alongside spiked ABA, influencing the baseline signal. Moreover, the refractive index (RI) of xylem sap differs significantly from that of the buffer, requiring reference and buffer correction. By measuring ABA concentration at the report point during the dissociation phase, when only buffer flows over the sensor surface, the SPR sensor isolates the signal from specifically bound ABA, minimizing the matrix interference. This strategy eliminates the need for a calibration curve in xylem sap, making the sensor more robust than ELISA, which is susceptible to both matrix effects and endogenous ABA levels, complicating curve development and absolute quantification, as shown in the literature [63,64,65]. Although both ELISA and UPLC-MS/MS remain widely used for ABA quantification, both technologies require extensive sample preparation, such as internal standard spiking or solvent extraction [21,66]. In contrast, the newly developed SPR immunosensor simplifies analysis by eliminating the need for pretreatment and requiring only 30 µL of xylem sap per measurement. This represents a significant improvement over ELISA, which typically requires ~200 µL to perform both specific and nonspecific signal measurements.

Xylem sap samples from tomato plants subjected to drought and high salinity stress were analyzed using the newly developed SPR Biacore™ immunosensor, alongside the established analytical reference method UPLC-MS/MS [14,15,17]. As shown in Figure 4, both techniques consistently detected elevated ABA levels in stressed plants compared to controls, aligning with ABA’s known role in abiotic stress responses [6,8,9]. Although the developed SPR Biacore™ immunosensor underestimated the absolute concentration values compared to UPLC-MS/MS, possibly due to calibration bias in both techniques or the specific detection of enantiomers by the antibody, the strong positive correlation indicates that it reliably reflects relative changes in concentration across samples. However, in contrast to the UPLC-MS/MS data, the SPR-derived values correspond well with literature ranges. For example, Albacete et al. reported xylem sap ABA concentrations of 5–10 ng/mL in control plants (cv. Moneymaker) and 25–30 ng/mL under salinity stress, slightly lower than the 40 ng/mL observed in our study [67]. Similarly, Kudoyarova et al. found xylem sap ABA levels ranging from 28 ng/mL to 58 ng/mL in tomato plants (cv. Ailsa Craig), under partial rootzone drying over two days [68]. The latter value is comparable to the concentrations detected here after 48 h of nutrient solution withdrawal. Despite methodological differences across studies, these comparisons confirm that our developed SPR immunosensor provides ABA quantification within expected physiological ranges.

Although the initial investment in the Biacore™ SPR system and sensor chips is high, this cost is balanced by the high signal-to-noise, minimal sample preparation requirements, and low consumption of reagent material. Furthermore, the SPR biosensor features a reusable CM5 chip, which, once functionalized with the antibody, enables multiple measurements without compromising stability or sensitivity, as shown in Supplementary Figure S6. Similarly, Guo et al. demonstrated long-term chip stability for their immunoassay using a CM7 chip over 125 cycles, despite requiring regeneration steps [69]. Although ELISA plates are cheaper, they are single-use, which limits their efficiency for repeated analysis. Moreover, the Biacore™ SPR system’s high degree of automation with quick two-minute measurements reduces manual labour and operator involvement, improving reproducibility and time efficiency. The platform also enables detailed characterization of the antibody–antigen interaction through affinity and kinetic rate constants, helping to select the best antibody for the bioassay. These advantages make the SPR platform a cost-effective, flexible, and efficient solution for high-throughput, high-precision hormone analysis in plant research. Alternatively, the bioassay concepts developed on the Biacore™ SPR system have the potential to be transferred to other, cheaper SPR-based biosensing platforms, such as fiber optic (FO)-SPR [70,71].

Future improvements in xylem sap sampling should prioritize plant viability while enabling location-specific and repeated sampling for ABA analysis [72,73]. Furthermore, it remains to be determined whether Biacore™ can reliably quantify ABA levels in more complex matrices such as plant extracts. Such advancements would further position the SPR biosensor as a diagnostic tool capable of capturing the dynamic, spatiotemporal patterns of ABA levels. In conclusion, this label-free, non-competitive SPR immunosensor represents a sensitive and efficient alternative to the conventional ELISA and UPLC-MS/MS techniques, and establishes itself as a valuable tool for advancing hormone analysis in plant stress physiology.

## Figures and Tables

**Table 1:** Average values of kinetic parameters (k_a_, k_d_, and K_D_) ± SD of the 1:1 binding model for the anti-ABA antibody interaction with ABA, ABA aldehyde, ABA-GE, and ACC (n = 4). ND = not detectable.

	ABA	ABA Aldehyde	ABA-GE	ACC
	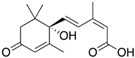	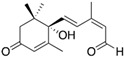	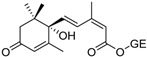	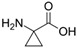
k_a_ (1/Ms)	7.86 ± 0.90 × 10^4^	4.82 ± 0.38 × 10^3^	1.97 ± 0.29 × 10^3^	ND
k_d_ (1/s)	3.69 ± 0.04 × 10^−2^	3.74 ± 0.11 × 10^−2^	3.09 ± 0.20 × 10^−2^	ND
K_D_ (M)	4.66 ± 0.73 × 10^−7^	8.12 ± 0.83 × 10^−6^	1.74 ± 0.31 × 10^−5^	ND
RU at 150 ng/mL	8.97 ± 0.42	1.91 ± 0.04	0.76 ± 0.09	ND

## Data Availability

The research data are available upon request.

## Supplementary Materials

The following supporting information can be downloaded at: https://www.mdpi.com/article/10.3390/bios15110725/s1, Figure S1: Absence of nonspecific binding of xylem sap matrix to the reference flow cell Fc1; Figure S2: Anti-ABA antibody immobilization graph and the residuals graph for the ABA and ABA-BSA sensorgrams; Figure S3: DMSO bulk shift correction for each ABA analogue analyte; Figure S4: Kinetic sensorgram, Rmax value, and residual data for each ABA analogue analyte; Figure S5: Comparison of the binding characteristics of ABA diluted in acetate buffer with ABA diluted in acetate buffer supplied with BSA; and Figure S6: Stability curves of channel 1 of the anti-ABA immunosensor chip on the Biacore™ SPR platform.

## Abbreviations

The following abbreviations are used in this manuscript:

ABA	Abscisic acid
ABA aldehyde	Abscisic aldehyde
ABA-BSA	Abscisic acid bovine serum albumin
ABA-GE	ABA glucosyl ester
ABI1	Abscisic acid insensitive 1
ACC	1-aminocyclopropane-1-carboxylic acid
C	Control plant sample
EC	Electrical conductivity
EDC	1-ethyl-3-(3-dimethylaminopropyl)carbodiimide
Fc	Flow cell
k_a_	Association rate constant
k_d_	Dissociation rate constant
K_D_	Equilibrium dissociation constant
LOD	Limit of detection
NHS	N-hydroxysuccinimide
PP2C	Protein phosphatase 2C
PYL1	Pyrabactin resistant 1 like 1
RI	Refractive index
SPR	Surface plasmon resonance
T	Treated plant sample
UPLC	Ultra-performance liquid chromatography
