# Supplementary material for: Label-Free Rapid Quantification of Abscisic Acid in Xylem Sap Samples Using Surface Plasmon Resonance
Source: Biosensors (Basel). 2025 Nov 1;15(11):725. doi: 10.3390/bios15110725 (PMC12650577; doi:10.3390/bios15110725)
Supplement: Supplementary file 1 [file biosensors-15-00725-s001.zip › biosensors-3887169-supplementary.pdf]

Supplementary information

Figure S1. Absence of nonspecific binding of xylem sap matrix to the reference flow cell Fc1

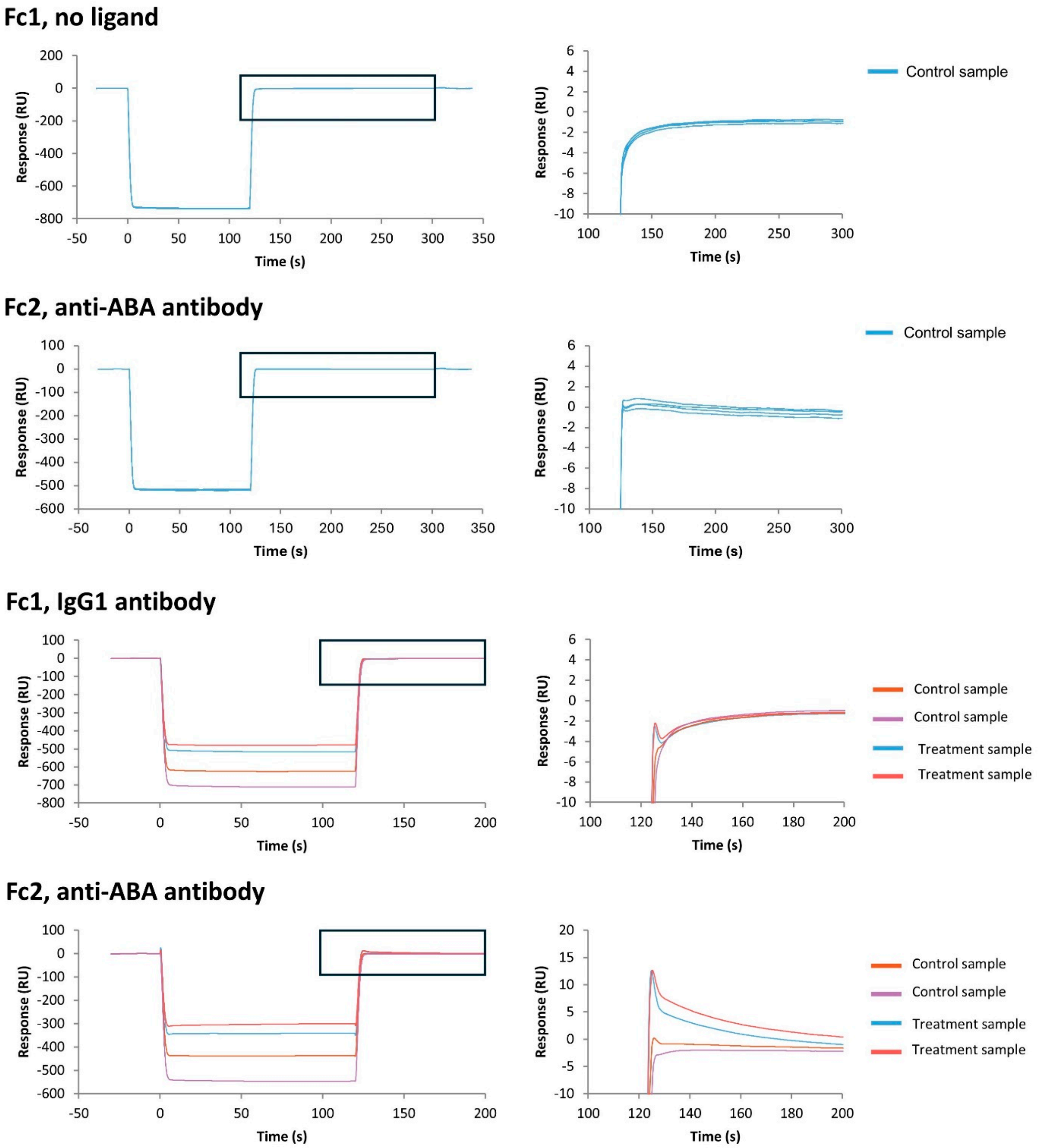

Figure S1: Nonspecific binding of various xylem sap samples, both from control and treatment plants, to the reference flow cell Fc1, either without a ligand or functionalized with an IgG1 antibody, on different chips, to mimic the anti-ABA antibody functionalized surface of Fc2, included to show specific binding signals. The IgG1 isotype antibody (cat. nr.: 555746; purchased from BD Biosciences (Erembodegem, Belgium)) was coupled to the sensor chip (20 µg/mL in 10 mM sodium acetate, pH 4.5).

Figure S2. Anti-ABA antibody immobilization graph and the residuals graph for the ABA, ABA-BSA, and BSA sensorgrams

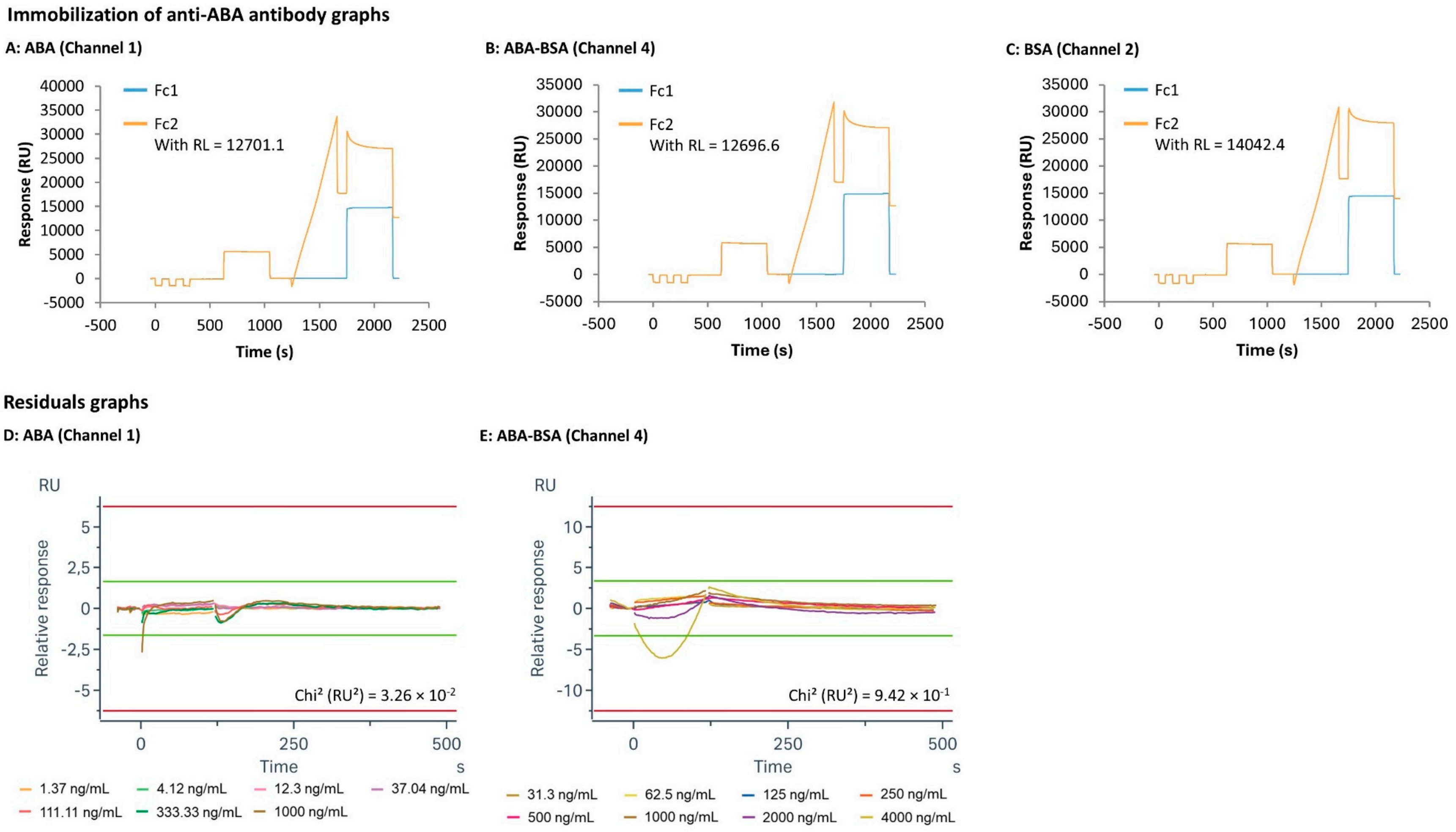

Figure S2: Sensorgrams of the anti-ABA antibody immobilization on Fc2 from: Channel 1 for ABA (A), Channel 4 for ABA-BSA (B), and Channel 2 for BSA (C), with the corresponding reference Fc1 and immobilization response level (RL); the residuals graphs for the sensorgrams, with the corresponding concentrations, of Channel 1 for ABA (D) and Channel 4 for ABA-BSA (E), with quality kinetic Chi². No fit was made for BSA, resulting in no residuals graph.

Figure S3. DMSO bulk shift correction for each ABA analog analyte

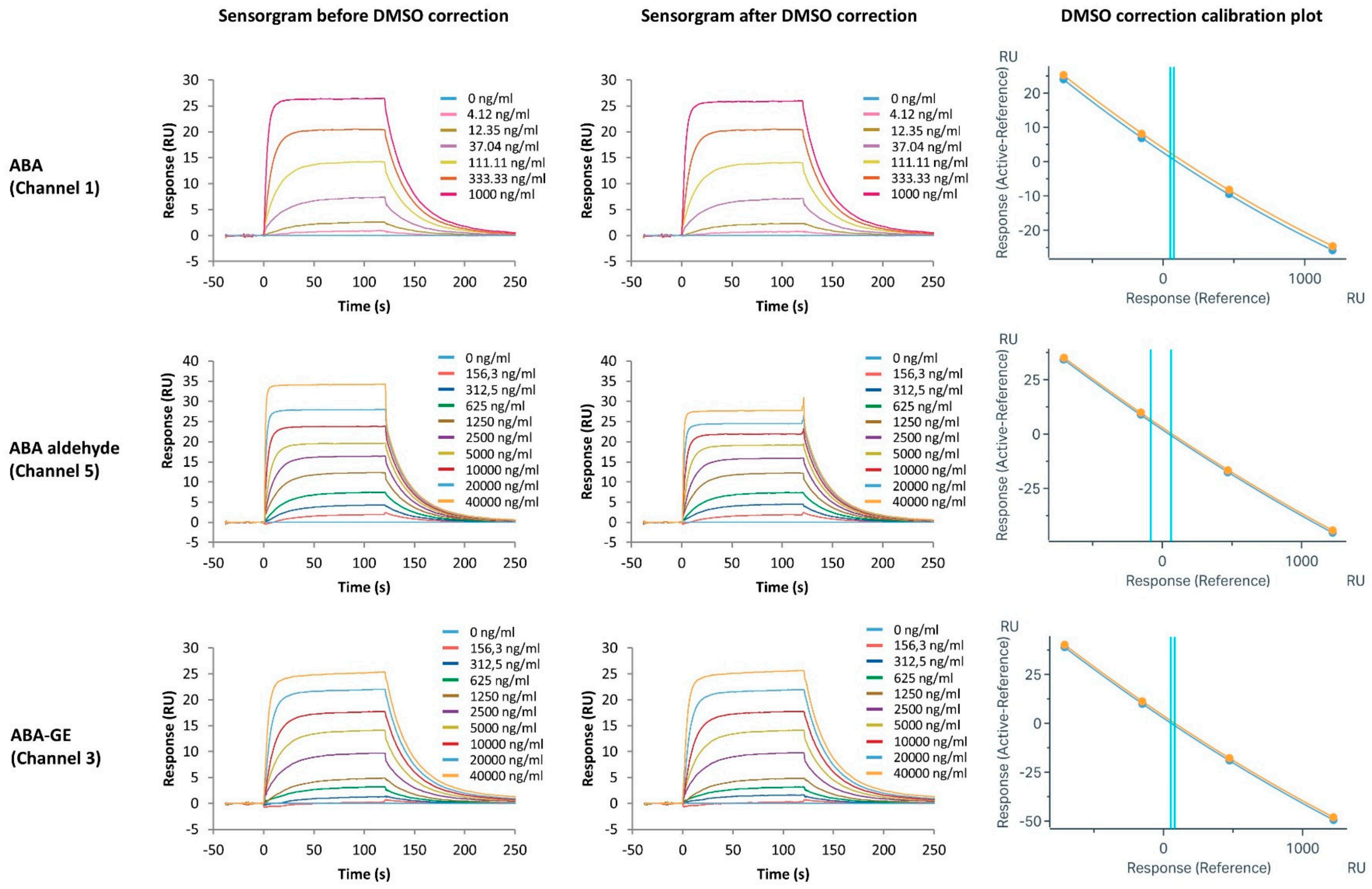

Figure S3: DMSO bulk shift correction of the ABA analogue data: ABA, ABA aldehyde, ABA-GE, including the sensorgrams before and after the correction, and the correction calibration plot with range of report points between blue lines.

Figure S4. Kinetic sensorgram, Rmax value, and residual data for each ABA analogue analyte

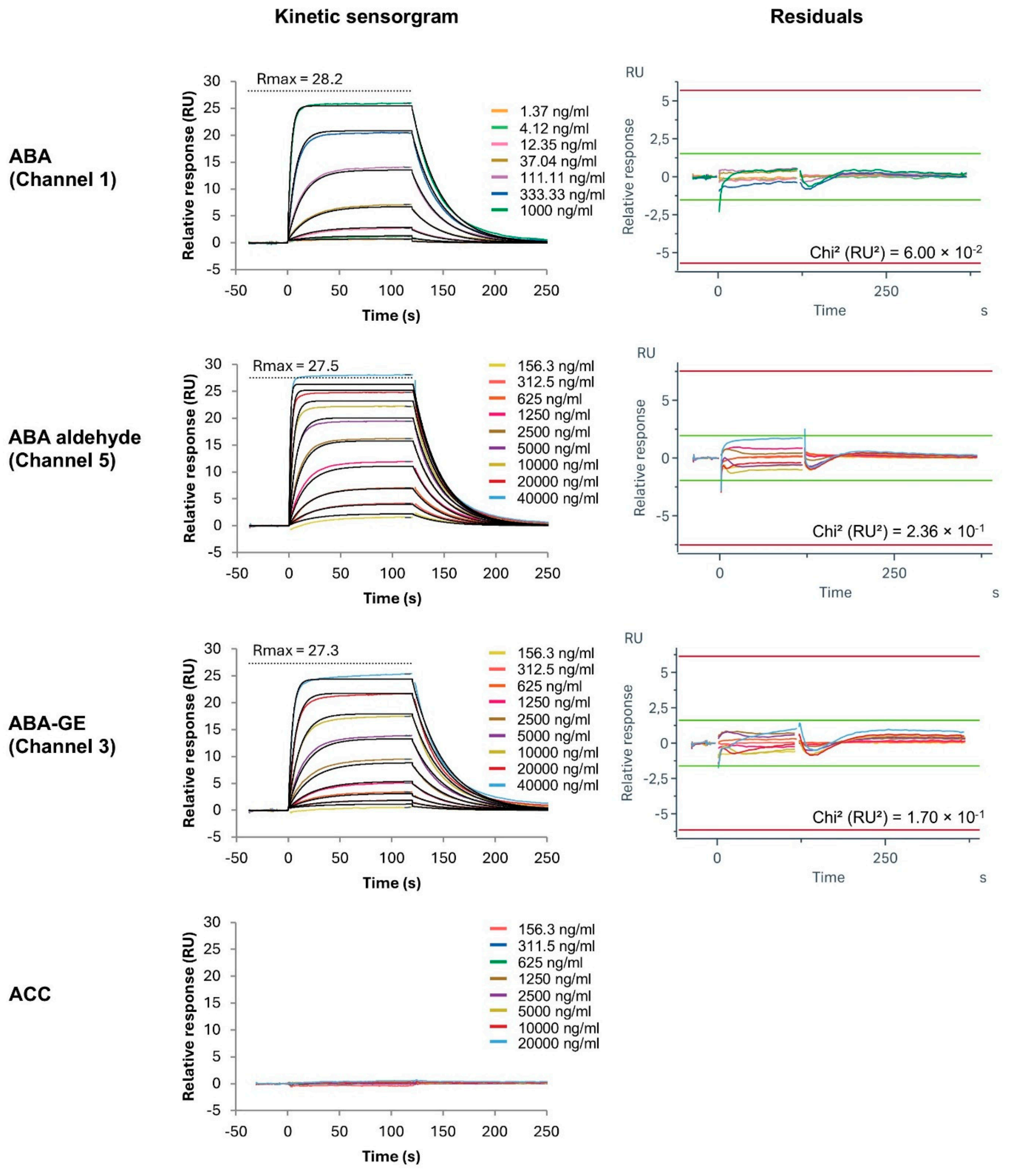

Figure S4: SPR sensorgrams (colored lines) and fitting curves (black lines), Rmax value, and residual data for each ABA analogue analyte: ABA, ABA aldehyde, ABA-GE and ACC. For ACC, there is no residuals graph as no fit was performed. No channel is indicated for ACC because the ACC concentrations were run in parallel mode over all 8 channels.

Figure S5. Comparison of the binding characteristics of ABA diluted in acetate buffer with ABA diluted in acetate buffer supplied with BSA

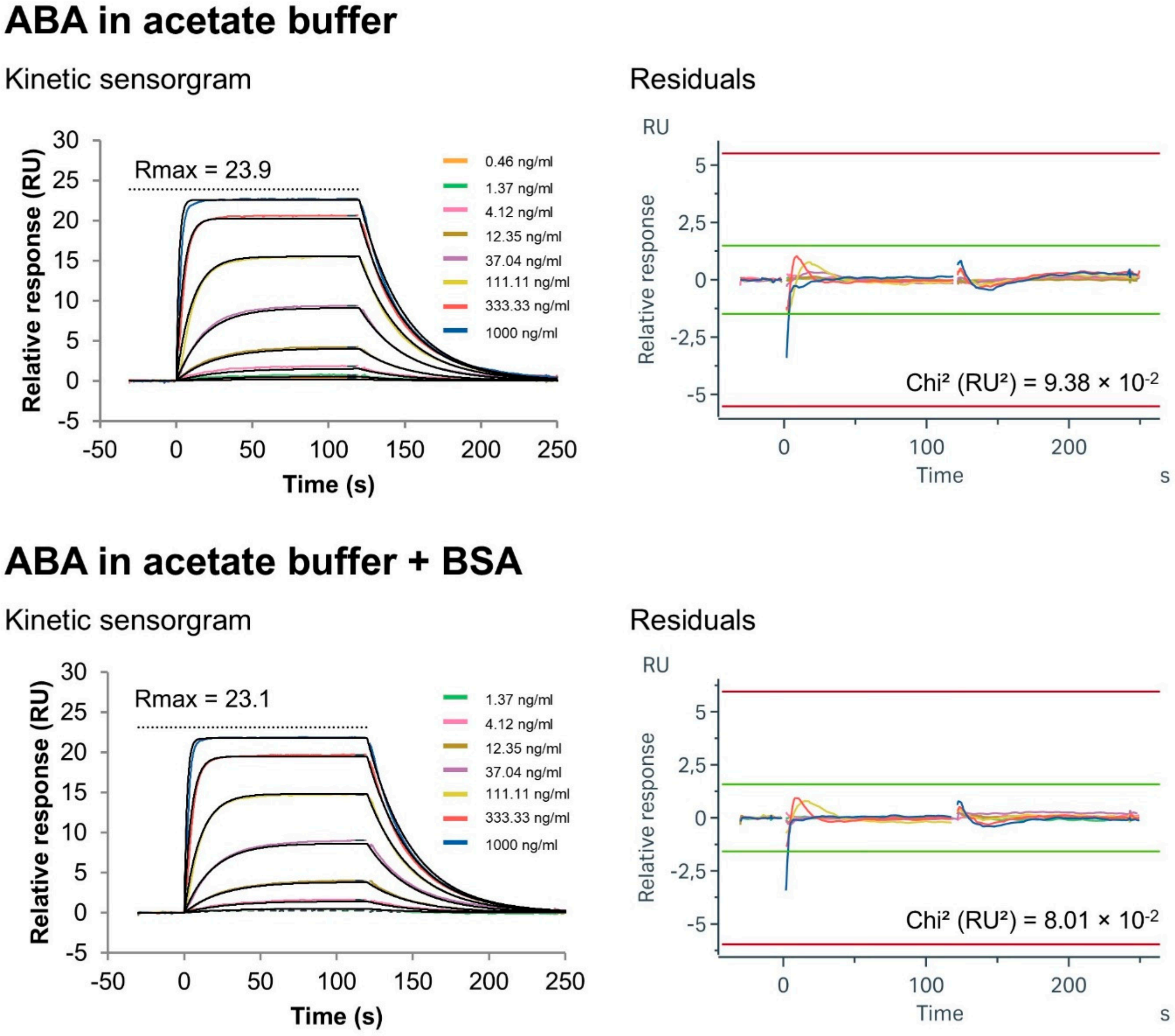

	**k_a (1/Ms)**	**k_d (1/s)**	**K_D (M)**
**ABA in acetate buffer**	1.48×10^5	3.39×10^{-2}	2.29×10^{-7}
**ABA in acetate buffer + BSA**	1.45×10^5	3.41×10^{-2}	2.35×10^{-7}

Figure S5: SPR sensorgrams (colored lines) and fitting curves (black lines), Rmax value, and residual data for the comparison of the binding characteristics of ABA diluted in acetate buffer with ABA diluted in acetate buffer supplied with BSA. For both, seven concentrations are shown for ABA (1000 ng/mL – 1.37 ng/mL in a three-fold dilution), with an additional concentration of 0.46 ng/mL for ABA in acetate buffer. The table below indicates the equilibrium dissociation constant (K_D) and the kinetic rate constants (k_a and k_d) of the 1:1 binding model (n=1).

Figure S6. Stability curves of channel 1 of the anti-ABA immunosensor chip on the Biacore™ SPR platform

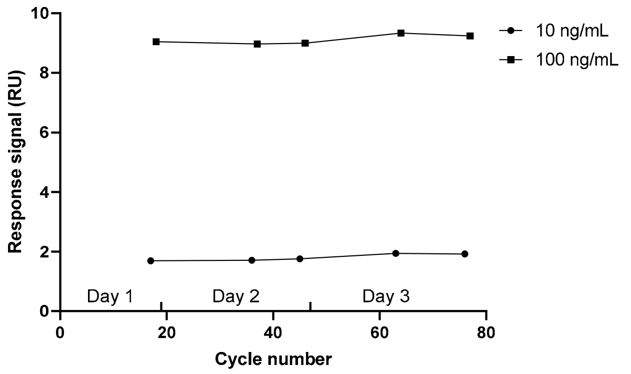

Figure S6: Stability of channel one of the anti-ABA immunosensor chip by repeatedly injecting 10 ng/mL (n=5) and 100 ng/mL (n=5) of ABA solutions and monitoring the response signal over the course of 3 days and 77 cycles.
